# Therapeutic Strategies for Modulating the Extracellular Matrix to Improve Pancreatic Islet Function and Survival After Transplantation

**DOI:** 10.1007/s11892-018-1014-4

**Published:** 2018-05-19

**Authors:** Alexandra M. Smink, Paul de Vos

**Affiliations:** 1 Department of Pathology and Medical Biology, Section of Immunoendocrinology, University of Groningen, University Medical Center Groningen, Hanzeplein 1, EA11, 9713 GZ Groningen, The Netherlands; 2 Department of Pathology and Medical Biology, University Medical Center Groningen, University of Groningen, Groningen, The Netherlands

**Keywords:** Pancreatic islets, Extracellular matrix, Islet transplantation, Type 1 diabetes

## Abstract

**Purposes of Review:**

Extracellular matrix (ECM) components modulate the interaction between pancreatic islet cells. During the islet isolation prior to transplantation as treatment for type 1 diabetes, the ECM is disrupted impacting functional graft survival. Recently, strategies for restoring ECM have shown to improve transplantation outcomes. This review discusses the current therapeutic strategies to modulate ECM components to improve islet engraftment.

**Recent Findings:**

Approaches applied are seeding islets in ECM of decellularized organs, supplementation of specific ECM components in polymeric scaffolds or immunoisolating capsules, and stimulating islet ECM production with specific growth factors or ECM-producing cells. These strategies have shown success in improving functional islet survival. However, the same experiments show that caution should be taken as some ECM components may negatively impact islet function and engraftment.

**Summary:**

ECM restoration resulted in improved transplantation outcomes, but careful selection of beneficial ECM components and strategies is warranted.

## Introduction

Transplantation of pancreatic islets in patients with type 1 diabetes results in stable glycemic control and the prevention of the development of secondary complications, such as cardiovascular diseases, nephropathy, and retinopathy [1]. However, the shortage of islet donors, the need for lifelong immunosuppression, and gradual decrease of islet function over time are still obstacles for large-scale application of this therapy [1]. Multiple factors contributing to graft failure have been identified including lack of adequate revascularization [2], reoccurrence of autoimmunity [3], the occurrence of an instant blood-mediated inflammatory response [4, 5], ischemic injury [6], and activation of NK(T) cells [7]. Also, damage to the extracellular matrix (ECM) induced during the enzymatic isolation of islets from the pancreas has been proposed as a factor influencing function and survival of islet grafts. However, only recently strategies have been proposed to overcome this damage to islet ECM prior to transplantation [8•, 9–11]. This review discusses the currently employed therapeutic strategies to modulate ECM components to protect and improve islet function and survival after transplantation. Also, we review how modulation of the ECM can have detrimental effects on functional survival of islets as we have the experience that ECM components or fragments might negatively impact islets or processes associated with engraftment.

## ECM Composition of Pancreatic Islets

The importance of ECM for islet function has been shown in several studies demonstrating its role in organizing the connections between endocrine cells, vascular endothelial cells, neural cells, and immune cells [12]. These interactions enable the rapid exchange of oxygen, nutrients, metabolites, signaling hormones, and of course islet hormones such as insulin and glucagon [13]. Pancreatic islets have an extensive network of ECM molecules [14–16], and these can be found in two distinct locations, the ECM in the basement membrane and in the interstitial ECM. In the basement membrane, the ECM is composed of a thin layer that separates islet cells from the exocrine cells and the blood vessels. This is the main target of enzymatic isolation of islets from the pancreas as it connects the endocrine and exocrine tissue. The interstitial ECM is a highly variable network of ECM proteins and polysaccharides in between the islet cells. In both the basement membrane and the interstitial ECM, the same type of ECM molecules can be found. All pancreatic ECM is composed of either glycosaminoglycans (GAGs) or fibrous proteins [17]. There are several types of GAGs: examples are heparan sulfate, chondroitin sulfate, dermatan sulfate, or keratan sulfate. One or more GAGs can bind to a core protein to form a proteoglycan. Proteoglycans form large complexes with other matrix components but can also bind water or growth factors [18]. GAGs are also involved in movement and stability of tissue [12, 19], but disturbances in their synthesis can also lead to islet amyloid formation and cellular dysfunction [20, 21].

Examples of fibrous ECM proteins in the pancreas are collagen, laminin, and fibronectin. The most abundant types of collagen in islets are collagen types I and IV, which are mostly found in the islet basement membrane [22]. They both regulate fibronectin by restraining cell-fibronectin interactions. Already in the fetal stage, collagens modulate cell-matrix interactions and development of the pancreas [12, 23]. Although it is less clear how collagen influences integrity of the mature pancreas, its abundance suggests that also in the adult pancreas, it is responsible for tissue integrity and cell interactions. Besides collagen, laminins are abundantly present in islets. Laminins bind to several different integrin and non-integrin receptors expressed by the islets to promote insulin secretion, gene expression, beta-cell survival, and proliferation [12, 24]. Furthermore, fibronectin is involved in adhesion and the binding of ECM molecules, but is also involved in connecting endocrine cells [12, 22]. Additionally, fibrin can be considered as part of the ECM. Fibrin is naturally formed from fibrinogen by the enzyme thrombin. The fibrin network provides anchor sites for growth factors and cells implicated in cell migration and tissue repair [17, 25].

ECM molecules bind to integrin receptors that are expressed by islets. These receptors modulate cell-cell and cell-ECM interactions to regulate functional islet survival [12]. There are 24 of these transmembrane receptors, which are formed by different combinations of integrin alpha and beta subunits. For instance, binding of laminin-5 to the α6β1 integrin receptor results in beta-cell proliferation and enhanced insulin secretion [26]. Additionally, binding of fibronectin, laminin, collagen type I, or collagen type IV to the α3β1 integrin receptor improves cell viability and proliferation of the beta-cell line INS-1 [27]. Besides integrin receptors, there are also non-integrin receptors that are involved in these interactions. Examples of non-integrin receptors are discoidin domain receptors (DDRs), laminin-receptor 1, dystroglycan protein complex, and Lutheran blood group glycoprotein [12]. Collagen type IV is one example that acts via DDRs and several types of laminin bind to the other non-integrin receptors [28, 29]. The described ECM effects appear to be mediated via both integrin and non-integrin receptors, but more research is needed to determine the exact signaling pathways.

During the enzymatic isolation of pancreatic islets prior to clinical transplantation, the ECM and the vasculature are disrupted. Currently, human islets are isolated by application of enzyme mixtures of collagenases [30]. There is laboratory-to-laboratory variation in the content of this enzyme mixture, but it contains collagenase, neutral protease, trypsin, and clostripain and it selectively breaks down the basement membrane that forms the connection between the exocrine and endocrine tissue [30]. However, these enzymes also damage the interstitial ECM. The enzyme mixture is injected via the pancreatic duct and damages several components of the islet ECM. It has detrimental effects on laminins [31], and it has been shown that the collagenases digest collagen types I, III, IV, and V [8•, 32, 33]. Furthermore, the enzyme mixture destroys the intracellular stores of GAG heparan sulfate [15]. Destruction of the ECM components leads to a decrease in cell viability and therefore also affects transplant outcomes.

## Therapeutic Strategies to Modulate and Restore Islet ECM

As restoring or supplementing ECM of islets might improve the outcome of islet transplantation, different strategies have been proposed for restoration of islet ECM. Some strategies coming from the tissue-engineering field, discussed below, might be beneficial for the islet field as well. In general, these strategies aim on modulating the ECM of islets to mimic the biochemical composition of the native pancreatic ECM, its structure, and its viscoelastic properties [34, 35].

### Decellularized ECM Scaffolds for Islet Transplantation

In the last few years, the use of ECM from decellularized organs has emerged in the field of tissue engineering and islet transplantation [36]. A major advantage of decellularized ECM structures is the major reduction of immunogenicity when all cellular materials are removed. The procedure leaves behind a scaffold of ECM components that can function as a support structure for transplanted cells. In theory, the pancreas would be the ideal organ to decellularize for islet transplantation since it will resemble the native pancreatic ECM. Several studies show that islet function and survival are maintained when cultured on decellularized pancreata [37–41], but in vivo data are absent up till now. More in vivo data is available on application of decellularized lung [42•] and pericardium [43•] as ECM scaffold for islet grafts. Abualhassan and coworkers infused 500 mouse islets into decellularized lung tissue and transplanted the grafts into the peritoneal cavity of diabetic mice [42•]. Normoglycemia was obtained in 67% of the mice, but only in 13% when islets were transplanted into the peritoneal cavity without an ECM scaffold. Furthermore, they demonstrate efficacy with human islets as well where diabetes reversal was demonstrated with 1000 human islet equivalents (IEQ) when transplanted into decellularized lung tissue. However, normoglycemia could not be maintained until the end of the study. Furthermore, Wang and coworkers tested the efficacy of decellularized pericardium and showed that with 250 syngeneic islets, 83% of diabetic mouse recipients became normoglycemic when transplanted in the epididymal fat pad [43•]. Even with a minimal number of 150 islets, diabetes was reversed in 47% of the mice. Blood glucose levels were stable during the whole study and normoglycemia was maintained until the end of the study, which was 300 days post transplantation.

### Artificial Replacement of ECM Components

Another therapeutic strategy to enhance functional survival of islets is by adding specific ECM molecules. The efficacy of such an approach has been shown in the field of immunoisolation by encapsulation of islets [8•, 9, 44, 45]. Encapsulation of islets in an immunoprotective and semipermeable membrane allows for successful transplantation of islets in the absence of immunosuppression [46]. To enhance longevity of encapsulated pancreatic islets in immunoprotective alginate-based microcapsules, single ECM molecules and their combinations have been tested [8•, 9]. The efficacy of such an approach was demonstrated, but it was also shown that a stepwise and careful method has to be chosen as not all ECM molecules are beneficial for islet function and concentrations that are too high may even kill islet cells. For example, excessively high concentrations of collagen IV had detrimental effects on glucose-induced insulin secretion [8•, 9]. Also, only the laminin sequences RGD, LRE, and PDSGR in combination with collagen IV had a positive impact on the function of human islets, and interestingly, islet function-promoting effects were laminin-type dependent. The three laminin sequences RGD, LRE, and PDGRS had a positive effect on glucose-induced insulin release of islets in vitro [8•]. All three molecules were also effective in reducing cytokine-mediated cell death in islet cells, whereas other laminin sequences did not have these effects. All combinations of collagen IV with either RGD, LRE, or PDGRS improved islet cell survival and reduced necrosis and apoptosis after interleukin-1β, interferon-γ, and tumor necrosis factor-α exposure [9]. However, there were also laminin-specific effects. Collagen IV-RGD and collagen IV-LRE reduced the release of danger-associated molecular patterns from islets, while collagen IV-PDGRS was ineffective. Collagen IV-RGD and collagen IV-PDSGR, but not collagen IV-LRE, reduced nitric oxide release from encapsulated human islets [9]. Moreover, the oxygen consumption rate of islets was only beneficially influenced by collagen IV-LRE and collagen IV-PDGRS and to a lesser extent by RGD inclusion [9]. Campanha-Rodrigues and coworkers conducted islet transplantations with laminin-1-alginate microcapsules [44]. Laminin-1 significantly improved the long-term survival and function of the islets compared with alginate capsules without laminin-1.

ECM components have also been tested in combination with polymeric scaffolds. These polymeric scaffolds are not immunoprotective but serve as a retrievable transplantation site for insulin-producing cells. This approach may be instrumental for replenishable cell sources such as stem cells that still suffer from issues making retrievability a mandatory criterion for human application [47]. In this setting, Naijar and coworkers tested a fibrin scaffold for islet transplantation under the skin and in the epididymal fat pad [48]. Vascular growth factors could easily be incorporated in these fibrin scaffolds resulting in improved vascularization, engraftment, and functional graft survival at both transplantation sites. The efficacy of fibrin as an islet scaffold has recently also been clinically demonstrated, as successful islet transplantation was performed in a fibrin scaffold placed in the omentum of a patient with type 1 diabetes (ClinicalTrials.gov number: NCT02213003) [49•]. After receiving 11,280 IEQ/kg, the patient became normoglycemic and insulin independent, which was maintained up to 12 months after transplantation.

Coating of scaffolds with collagen IV can induce faster restoration of normoglycemia compared to islet transplantation in untreated scaffolds [50]. The collagen IV coating promoted islet cell viability and decreased islet apoptosis, which lead to an enhanced islet-metabolic function. Similar beneficial effects have been shown for laminins [51]. Coating scaffolds with the human laminin sequence 332 improved insulin secretion in response to glucose stimulation. Furthermore, Beenken-Rothkopf and coworkers supplemented hydrogel scaffolds with collagen type IV, fibronectin, and laminin [52]. These scaffolds supported function and survival of cells in vitro. In addition, our group performed several studies in which polymeric scaffold were supplemented with ECM. In these studies, fibrin was used to fill the pores of a porous polymer scaffold [2]. After 4 weeks of prevascularization under the skin of diabetic nude mice, transplantation of 800 islets resulted in diabetes reversal in 80% of the mice, while transplantation of islets without a scaffold never resulted in normoglycemia.

### In vivo Stimulation of ECM Production

Approaches to restore the whole islet ECM are also being investigated, as an alternative to the supplementation of ECM components. In the native pancreas, ECM can be produced by fibroblasts in and around the islets. Co-incubation of fibroblast and islets results in improved functional survival of islets and a well-preserved ECM including high amounts of fibronectin [53]. Also, mesenchymal stromal cells (MSCs) have shown to have such beneficial effects on islets due to the secretion of fibronectin and laminin [54, 55]. An in vivo study showed that co-transplantation of MSCs and syngeneic islets under the kidney capsule of diabetic mice improved islet function and survival in the early post-transplantation period [56]. An advantage of this approach is that not only one component of the ECM is restored, but also multiple components are modulated which will be a better representation and restoration of the native ECM.

Another approach, not tested yet with islets but successful in other fields, is to restore the ECM in vivo by injecting factors that stimulate the fibroblasts and other cells around the transplanted islets to produce ECM components. Transforming growth factor (TGF)-β is important for tissue repair after injury and might be a suitable candidate [57]. TGF-β stimulates the proliferation of fibroblasts and the production of fibronectin and collagen [57]. Connective tissue growth factor might be another candidate since it is known to stimulate ECM production [58]. Up to now, these approaches are only tested in vitro and not extensively within the context of pancreatic islets. Some unconventional methods were also investigated, for example, high frequency vibrations to enhance TGF-β production of fibroblasts in vitro leading to increased production of fibronectin and collagen I [59].

## Potential Detrimental Effects of ECM

**Fig. 1:**
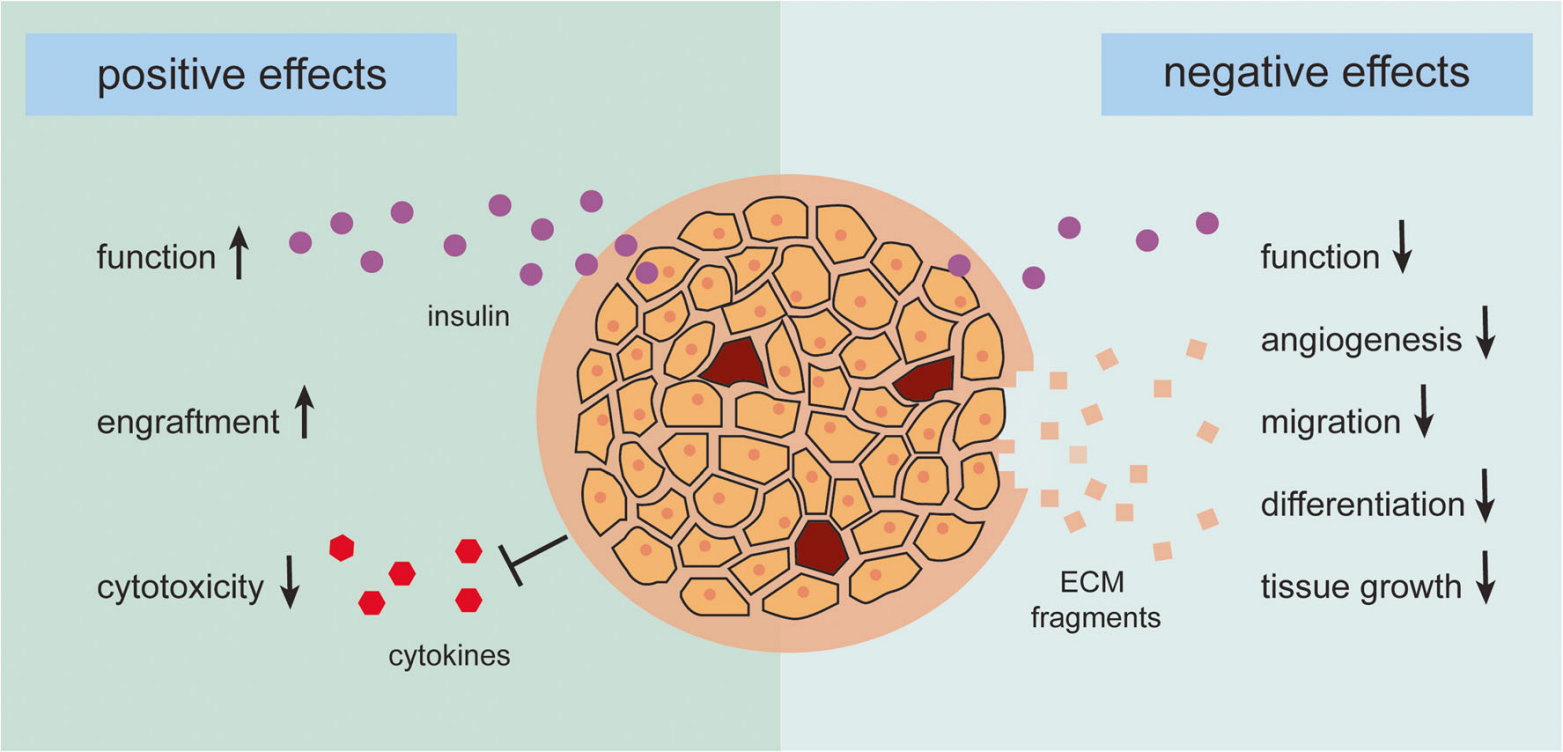
The positive and negative effects of modulating the extracellular matrix (ECM) of pancreatic islets. The enzymes used for isolating islets from the pancreas damage the native ECM of the islets influencing functional graft survival. Recently, strategies for restoring ECM have shown to improve islet function and engraftment and reduce sensitivity for cytotoxicity of cytokines resulting in better transplantation outcomes. The currently confirmed positive effects of restoring ECM before islet transplantation are depicted on the left side of the figure. For example, the laminin sequences RGD, LRE, and PDSGR have shown to improve the glucose-induced insulin response and thereby the function of beta cells, whereas addition of collagen IV or the laminin sequences RGD and PDSGR to the isolated islets protects them from cytokine-mediated cell death. However, research has also shown that restoring ECM can have negative effects on islets, which is depicted on the right side of the figure. For example, high concentrations of some ECM components, such as collagen IV, can be detrimental for islet function, and proteolytic fragments of ECM components are known to have adverse effects on processes involved in engraftment; they can inhibit angiogenesis, migration, differentiation, and tissue growth

It has been shown that ECM restoration results in reduced cytotoxicity and improved islet function [2, 8•, 9, 42•, 43•, 45, 49•]; caution is also warranted as modulation of the ECM can also have detrimental effects on functional survival of islets (Fig. 1). It is not sufficiently taken into account that not all ECM molecules will be beneficial for engraftment. We have shown that high concentrations of collagen IV hamper islet function [8•, 9] and some proteolytic fragments of ECM components are known to have adverse effects on processes involved in engraftment. These fragments inhibit angiogenesis, migration, differentiation, and tissue growth [60]. For example, the non-collagenous fragments of several collagen types such as collagen α2 (IV), collagen α3 (IV), collagen α6 (IV), and collagen α1 (XV) have anti-angiogenic effects and inhibit tumor growth [60, 61]. Due to these properties, ECM fragments are used as anti-tumor treatment [62].

## Future Perspectives

Many strategies discussed in this review have shown to be effective in vitro. However, more in vivo data should be obtained for adequate translation of feasibility in humans. ECM modification by using decellularized organs has currently shown promising in vivo results [42•, 43•]. Studies in larger animal models, such as pigs or non-human primates, should give more insight in which organ should be used for decellularization and if it is applicable in humans. Transplantation of islets in a fibrin scaffold has already shown to be beneficial in the clinic [49•]. Although beneficial effects have been described, further understanding of the exact mechanisms underlying the beneficial effects of ECM molecules, integrin receptors, and pathways is needed, as it also has been shown that ECM effects on pancreatic islets are ECM-type dependent. Besides ECM components, the integrin and non-integrin receptors regulate the interaction between islets and the ECM. The implications of isolation-related integrin damage should also be further investigated.

## Conclusion

In this review, we have discussed current strategies to improve islet transplantation outcomes by modulating the ECM. Modulation of the ECM has been shown to be an efficacious approach to enhance pancreatic islet function and survival and thereby improve transplantation outcomes. However, we also note that caution is warranted as ECM manipulation might also negatively impact islet function and/or engraftment. More systematic research is needed to provide a clinically applicable strategy to improve and restore the ECM in islets for transplantation and cure of patients with type 1 diabetes.
